# Hyaluronic Acid-Based Hydrogel Coating Does Not Affect Bone Apposition at the Implant Surface in a Rabbit Model

**DOI:** 10.1007/s11999-017-5310-0

**Published:** 2017-03-16

**Authors:** W. Boot, D. Gawlitta, P. G. J. Nikkels, B. Pouran, M. H. P. van Rijen, W. J. A. Dhert, H. Ch. Vogely

**Affiliations:** 1 Department of Orthopaedics, University Medical Center Utrecht, Utrecht, The Netherlands; 2 Department of Oral and Maxillofacial Surgery & Special Dental Care, University Medical Center Utrecht, Utrecht, The Netherlands; 3 Department of Pathology, University Medical Center Utrecht, Utrecht, The Netherlands; 4 Faculty of Veterinary Medicine, Utrecht University, Utrecht, The Netherlands

## Abstract

**Background:**

Uncemented orthopaedic implants rely on the bone-implant interface to provide stability, therefore it is essential that a coating does not interfere with the bone-forming processes occurring at the implant interface. In addition, local application of high concentrations of antibiotics for prophylaxis or treatment of infection may be toxic for osteoblasts and could impair bone growth.

**Questions/Purposes:**

In this animal study, we investigated the effect of a commercially available hydrogel, either unloaded or loaded with 2% vancomycin. We asked, does unloaded hydrogel or hydrogel with vancomycin (1) interfere with bone apposition and timing of bone deposition near the implant surface; and (2) induce a local or systemic inflammatory reaction as determined by inflammation around the implant and hematologic parameters.

**Methods:**

In 18 New Zealand White rabbits, an uncoated titanium rod (n = 6), a rod coated with unloaded hydrogel (n = 6), or a rod coated with 2% vancomycin-loaded hydrogel (n = 6) was implanted in the intramedullary canal of the left tibia. After 28 days, the bone volume fraction near the implant was measured with microCT analysis, inflammation was semiquantitatively scored on histologic sections, and timing of bone apposition was followed by semiquantitative scoring of fluorochrome incorporation on histologic sections. Two observers, blinded to the treatment, scored the sections and reconciled their scores if there was a disagreement. The hematologic inflammatory reaction was analyzed by measuring total and differential leukocyte counts and erythrocyte sedimentation rates in blood. With group sizes of six animals per group, we had 79% power to detect a difference of 25% in histologic scoring for infection and inflammation.

**Results:**

No differences were found in the amount of bone apposition near the implant in the No Gel group (48.65% ± 14.95%) compared with the Gel group (59.97% ± 5.02%; mean difference [MD], 11.32%; 95% CI, −3.89% to 26.53%; p = 0.16) or for the Van2 group (56.12% ± 10.06%; MD, 7.46; 95% CI, −7.75 to 22.67; p = 0.40), with the numbers available. In addition, the scores for timing of bone apposition did not differ between the No Gel group (0.50 ± 0.55) compared with the Gel group (0.33 ± 0.52; MD, −0.17; 95% CI, −0.86 to 0.53; p = 0.78) or the Van2 group (0.83 ± 0.41; MD, 0.33; 95% CI, −0.36 to 1.03; p = 0.42). Furthermore, we detected no differences in the histopathology scores for inflammation in the No Gel group (2.33 ± 1.67) compared with the Gel group (3.17 ± 1.59; MD, 0.83; 95% CI, −0.59 to 2.26; p = 0.31) or to the Van2 group (2.5 ± 1.24; MD, 0.17; 95% CI, −1.26 to 1.59; p = 0.95). Moreover, no differences in total leukocyte count, erythrocyte sedimentation rate, and neutrophil, monocyte, eosinophil, basophil, and lymphocyte counts were present between the No Gel or Van2 groups compared with the Gel control group, with the numbers available.

**Conclusion:**

The hydrogel coated on titanium implants, unloaded or loaded with 2% vancomycin, had no effect on the volume or timing of bone apposition near the implant, and did not induce an inflammatory reaction in vivo, with the numbers available.

**Clinical relevance:**

Antibiotic-loaded hydrogel may prove to be a valuable option to protect orthopaedic implants from bacterial colonization. Future clinical safety studies will need to provide more evidence that this product does not impair bone formation near the implant and prove the safety of this product.

## Introduction

Numerous approaches for locally applying antibacterial agents are being considered to try to minimize the risk of implant-related infection in the clinic. For example, bone cement in patients receiving cemented THA or TKA often is loaded with antibiotics [21]. Furthermore, a tibia nail coated with gentamicin-loaded polymer poly(D,L-lactide) for surgical treatment in closed or open tibial fractures, and in revisions, was associated with good clinical, laboratory, and radiologic outcomes after 6 months of followup in patients [6]. However, cementless THA or TKA currently lack options for local application of antibiotics. As it is important to minimize the risk of infection, there is a need to research alternative strategies to decrease the risk of infection for uncemented implants.

Ideally, a method for local prophylaxis of cementless implants should be biocompatible and should not interfere with bone apposition. One highly promising method for local prophylaxis of uncemented implants is using a resorbable, biocompatible hydrogel as a carrier for agents of interest [17]. Hydrogels generally offer easy application, flexibility in choice of antimicrobial agents, and complete resorption of the hydrogel [8, 11, 13, 19].

In previous studies, the commercially available hydrogel DAC^®^ (Defensive Antibacterial Coating; Novagenit Srl, Mezzolombardo, Italy) was shown to exert an antibacterial effect when loaded with antibiotics in vitro and in vivo [3, 7]. Various compounds, for example, vancomycin, gentamicin, or N-acetylcysteine, can be released from this hydrogel within 96 hours, with a release peak during the first 2 hours in vitro [3]. Further, the hydrogel loaded with 2% or 5% vancomycin was shown to be effective in reducing the local bacterial load in a rabbit implant-related infection model [7]. The hydrogel has been shown to be capable of resisting removal during implant insertion when used as a press-fit implant coating on uncemented femoral stems [3].

A local coating on uncemented implants should not interfere with the bone apposition near the implant surface, as this is an important feature for mechanical stability. In addition, local application of high concentrations of antibiotics may be toxic for osteoblasts and could impair bone growth [2, 4]. In previous work, histomorphometric evaluations showed no differences between the hyaluronic acid-based hydrogel coating and HYALGAN^®^ hydrogel (Fidia Farmaceulici s.p.a, Abano Terme, Italy) on cortical bone thickness, 12 weeks after application in a rabbit femur [7]. In the current study, we wished to investigate the effect of the hydrogel, either unloaded or loaded with 2% vancomycin, on bone apposition and timing of bone deposition near the implant surface and the effect on inflammatory parameters in a rabbit model.

Therefore, in the current rabbit implant-model study, we asked whether a hyaluronic acid-based hydrogel coating, either empty or loaded with 2% (v/w) vancomycin: (1) interferes with bone apposition and timing of bone deposition near the implant surface; and (2) induces a local or systemic inflammatory reaction as determined by inflammation around the implant and hematologic parameters.

## Materials and Methods

### Experimental Design

**Fig. 1:**
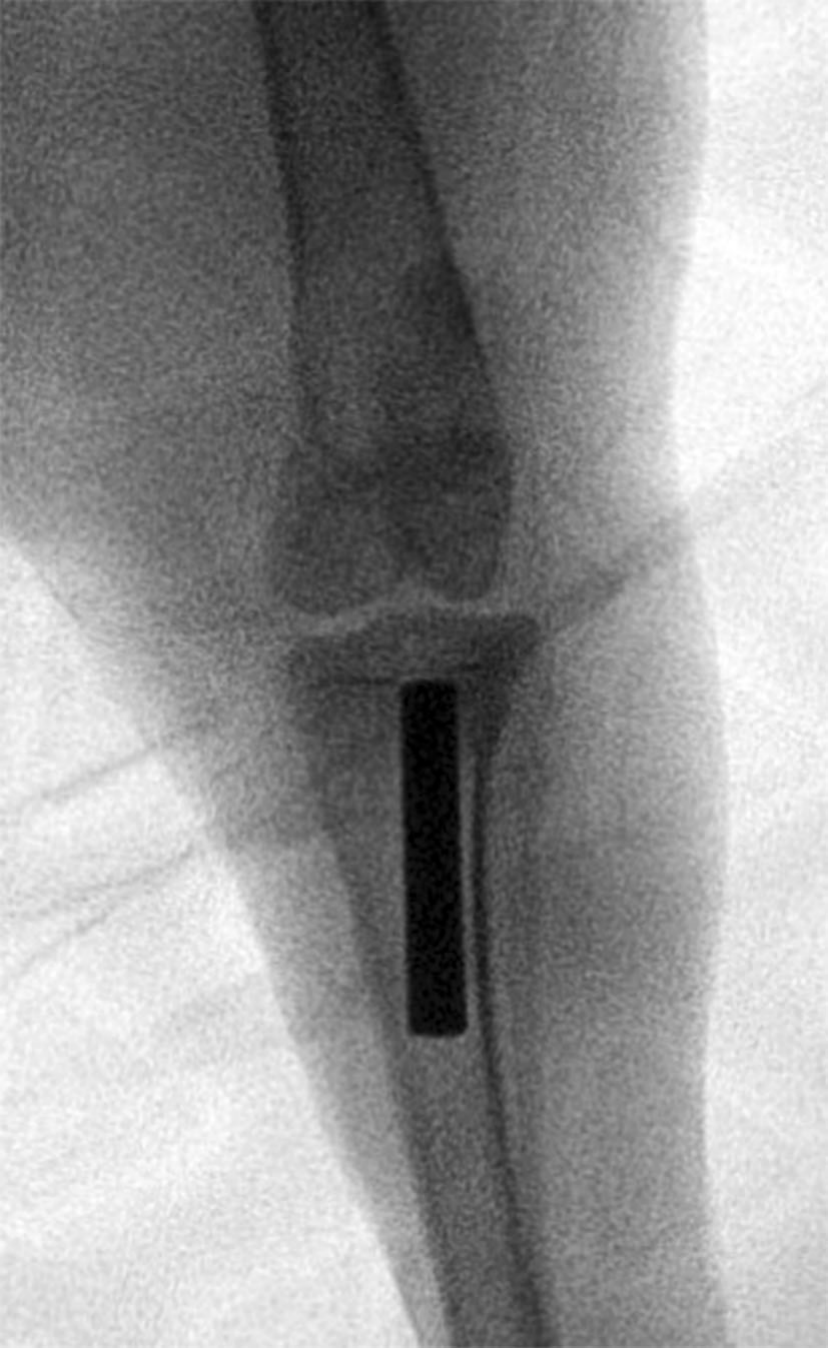
All animals received a titanium rod (diameter 4 mm; length 25 mm) in the intramedullary canal of the left tibia. Shown here is an AP radiograph of the knee obtained immediately after implantation of the implant.

An animal study with 18 rabbits was performed to evaluate the effect of implant coating on osseointegration and hematologic parameters. For this purpose an established in vivo implant-model was used [24]. Briefly, all animals received a titanium implant unilaterally in the left tibial intramedullary canal (Fig. 1). Fluorochromes were administered at Days 3, 7, and 21 to analyze the timing of bone formation. After 28 days, the animals were euthanized and explantation of the tibia and rod was done for microCT and histopathologic analyses. Blood was drawn preoperatively and weekly after implantation for hematologic analyses. Three groups (n = 6) were included: the hydrogel alone or loaded with 2% vancomycin, coated on the implant, were compared with an uncoated implant. The antibiotic vancomycin was chosen because it is effective against Gram-positive bacteria such as Staphylococci and Streptococci [10, 12], which account for more than 2/3 of prosthesis-related infections [20], and is a frequently used antibiotic in bone cement for local infection prophylaxis [21].

### Animals, Welfare, and Housing

The study was conducted with permission from the local ethics committee for animal experimentation in Utrecht, the Netherlands. Female New Zealand White (NZW; Charles River, L’Arbresle, France) rabbits were ordered at an age of 16 weeks and were allowed to acclimate for 12 to 14 days before surgery. The rabbits were housed in pairs, except for 2 to 3 days postoperatively until the surgical wounds were properly closed. Water was available ad libitum and the rabbits received 100 g of food (Stanrab; SDS, Essex, England) daily. The humane endpoint was defined as when the rabbits would lose weight exceeding 15% in 2 days or when they would experience shock or sepsis.

### Implants and Hydrogel Coating

The average surface roughness of the sandblasted titanium rods (Adler Ortho srl, Milan, Italy) (diameter 4 mm; length, 25 mm) was 5.6 µm. The surface roughness of the rods was comparable to that of uncemented femoral stems used for clinical purposes (Recta; Adler Ortho srl). DAC^®^, a patented hydrogel (Novagenit^®^) was used as a local carrier for vancomycin on the implant. Before implantation, the implants were not coated (No Gel group), coated with hydrogel (Gel group), or with hydrogel loaded with 2% (w/v) vancomycin (vancomycin hydrochloride; Hospira Benelux BVBA, Brussels, Belgium) (Van2 group). The hydrogel was provided as a sterile powder (60 mg) in a syringe and was reconstituted during surgery by mixing the powder with 1 mL sterile demineralized water, resulting in a solution with a concentration of 6% (w/v) hydrogel. In the Van2 group, the vancomycin was dissolved in the water before mixing with the hydrogel powder. The hydrogel was applied perioperatively on the surface of the titanium rods, using a spreader attached to the syringe with the hydrogel. The hydrogel was spread evenly on the complete surface of the titanium rod, after which the rod was immediately implanted.

### Surgery, Analgesia, and Anesthesia

Surgery was performed under aseptic conditions. Preoperatively, the animals received subcutaneous buprenorphine hydrochloride (0.03 mg/kg, Temgesic^®^; RB Pharmaceuticals Limited, Slough, United Kingdom) for analgesia. Anesthesia was initiated by subcutaneous injections of ketamine (10–15 mg/kg; Narketan^®^ 10; Vétoquinol BV, ‘s-Hertogenbosch, the Netherlands) and Dexdomitor^®^ (0.15–0.25 mg/kg; Orion Corporation, Espoo, Finland). Anesthesia was maintained by an intravenous line of 1:10 Dexdomitor^®^ and ketamine in NaCl.

Before the first incision, the hair of the left hind leg was removed and the skin was disinfected with 10% povidone-iodine (Betadine^®^ solution; Meda Pharma BB, Amstelveen, the Netherlands). The knee was opened with a medial parapatellar incision. Anterior to the cruciate ligaments, the tibial intramedullary canal was opened with an awl and reamed with a drill (diameter, 4.1 mm). Next, the implant, with or without hydrogel was implanted. The joint and skin were closed with Vicryl^®^ size 3-0 (Ethicon Inc, Johnson & Johnson, Peterborough, Ontario, Canada) and Monocryl^®^ size 3-0 (Ethicon), respectively. Radiographs were taken to verify the position of the implants in the proximal intramedullary tibial cavity and to verify an undamaged cortex. Anesthesia was reversed with Atipam^TM^ (0.5–1.0 mg/kg; Eurovet Animal Health BV, Bladel, the Netherlands). Postoperative analgesia with buprenorphine hydrochloride (0.03 mg/kg, subcutaneous) was administered every 8 hours for 48 hours.

### Fluorochrome Administration

Fluorochrome labels can be incorporated at sites of mineralization of bone and labels the front of mineralization at the time of administration [22]. By administering the labels at different times, bone formation can be followed with time. To observe bone apposition in the current study, rabbits were injected with two fluorochrome labels: xylenol orange (Xylenol Orange tetrasodium salt, 398187; Sigma-Aldrich, St Louis, MO, USA) and calcein green (Calcein disodium salt, 21030; Sigma-Aldrich). Two different administration schedules were used for analysis of early and late fluorochrome deposition. In each group, half of the animals were injected on Days 3 and 10 and the other half were injected on Days 7 and 21 with xylenol orange and calcein green respectively.

### Postoperative Followup and Euthanasia

Blood was collected preoperatively and weekly thereafter for analyses of total and differential leukocyte counts (neutrophils, monocytes, eosinophils, basophils, and lymphocytes), and erythrocyte sedimentation rates (ESRs). The analyses were performed by the Department of Clinical Chemistry and Haematology (UMC Utrecht, The Netherlands). The animals were euthanized 28 days after surgery with an overdose of intravenous sodium pentobarbital (Euthanimal^®^ 40%; Alfasan Nederland BV, Woerden, the Netherlands), after inducing general anesthesia. This time was chosen to be able to detect differences between the groups during an early phase of bone formation.

### Postmortem Sample Acquisition and Analyses

The operative areas were depilated and disinfected with 10% povidone-iodine. The proximal tibiae were explanted under sterile conditions with a saw (Dremel^®^ Model 300; Dremel Europe, Breda, the Netherlands) and placed in 10% formalin.

For microCT imaging for bone volume fraction analysis, all samples (six per group) were scanned after fixation with formalin using a microCT scanner (Quantum FX MicroCT; Perkin Elmer, Waltham, MA, USA) with a voxel size of 60 × 60 × 60 μm^3^. The images were reconstructed automatically in three dimensions using the built-in microCT software (Analyze 11.0). Bone apposition near the implant was measured as the percentage of bone volume within a distance of 180 µm from the entire cylindrical surface (excluding the ends) of the implant.

For histopathology and fluorochrome analyses, the tibia containing the implant was embedded after performing microCT. After fixation, the samples were dehydrated through a graded ethanol series and embedded in methylmethacrylate. Per mL, the methylmethacrylate solution consisted of 0.8 mL methylmethacrylate (Merck KGaA, Darmstadt, Germany), 0.2 mL Plastoid^®^-N (Sigma-Aldrich Chemie GmbH, Steinheim, Germany), and 28 mg benzoyl peroxide (Sigma-Aldrich, St Louis, MO, USA). After methylmethacrylate polymerization, sections of 20 to 30 µm were cut on a microtome (Leica SP1600; Leica Biosystems Nussloch GmbH, Nussloch, Germany). Per animal, two sections (one distal and one proximal section) of the left tibia with the rod were made for histopathology and two sections were made for fluorochrome analysis.

For histopathology, the sections were stained with 1% methylene blue solution and subsequently with 0.3% basic fuchsin solution. For semiquantitative scoring of inflammation, the scoring system by Vogely et al. [24] was used. This system quantifies 11 inflammation parameters which results in a score between 0 (no reaction) and 56 (serious reaction). Per animal, two sections (one distal and one proximal section) were scored. Two observers (WB and PGJN), blinded to the treatment, scored the sections and reconciled their scores if there was disagreement.

To examine the timing of bone apposition on the implant surface, unstained sections were analyzed microscopically for the presence of fluorochromes adjacent to the implant. If a particular fluorochrome was present a score of 1 was given, if there was no bone or fluorochrome present around the implant, a score of 0 was given. Scoring was performed by two observers blinded to the treatment (WB and MHPvR), and they reconciled their scores if there was disagreement. Scores were averaged per group. As two different schedules were used for administering fluorochromes, three animals were included for each time. One proximal and one distal section were scored per animal. Results were incomplete as one rabbit from the No Gel group was not injected with xylenol orange on Day 3. Furthermore, calcein green was not administered to six of nine rabbits on Day 10, therefore the results of these animals were excluded from further analysis.

### Statistical Analyses

With the group sizes used in our study, we had 79% power to detect a difference of 25% in histologic scoring for infection and inflammation at a probability less than 0.05 [24]. This outcome parameter was used for the power calculation as no existing data for bone-volume fraction for this specific animal model were available. The results from the microCT, histopathologic analyses, and the blood values for each time were compared by a one-way ANOVA with Dunnett’s post hoc test, including the No Gel group as the control. All statistical calculations were performed using SPSS Version 20.0 (IBM Corp, Armonk, NY, USA). A probability value less than 0.05 was considered significant and results are presented as mean (± SD), mean difference (MD), and 95% CI.

## Results

### Bone Apposition and Timing of Bone Deposition Near the Implant Surface

#### Bone Apposition

**Fig. 2A–B:**
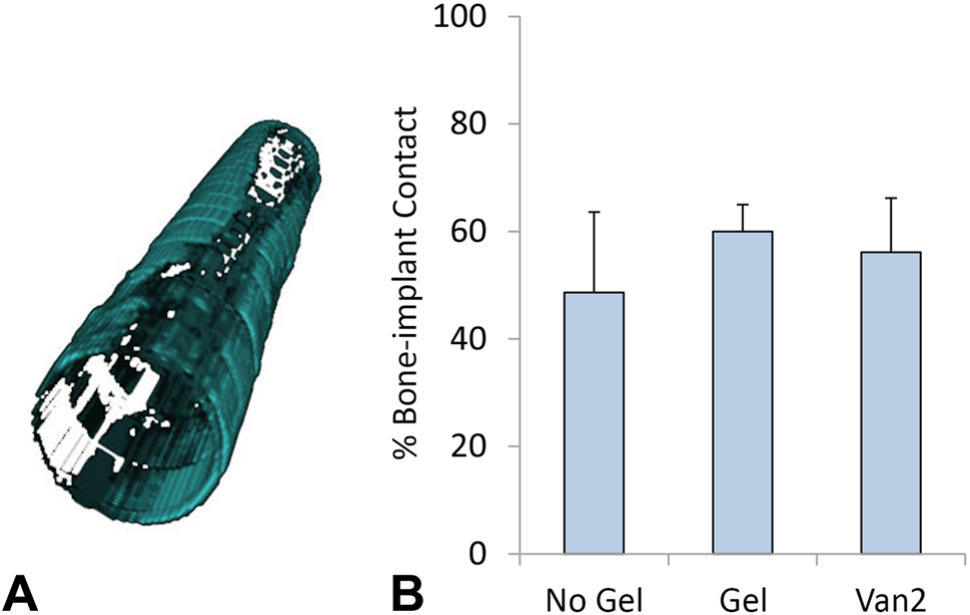
The bone volume fraction percentages near the implant surface were measured for all groups using microCT. **(A)** The bone present within 180 µm from the implant surface is shown in blue-green. **(B)** No differences were found in the bone volume percentages near the implant of the Gel or Van2 groups compared with the No Gel group. Data are shown as mean and SD.

All three groups showed bone volume fraction percentages near the implant surface between 49% and 60% (Fig. 2A). The bone volume fraction percentage in the No Gel group was 48.65% ± 14.95% (Fig. 2B). With the numbers available, no differences were found in bone volume fraction percentages for the Gel group (59.97% ± 5.02%; MD, 11.32%; 95% CI, −3.89% to 26.53%; p = 0.16) or for the Van2 group (56.12% ± 10.06%; MD, 7.46%; 95% CI, −7.75% to 22.67%; p = 0.40) compared with the No Gel group.

### Histologic Analysis of Fluorochrome Incorporation for Timing of Bone Deposition

**Fig. 3A–B:**
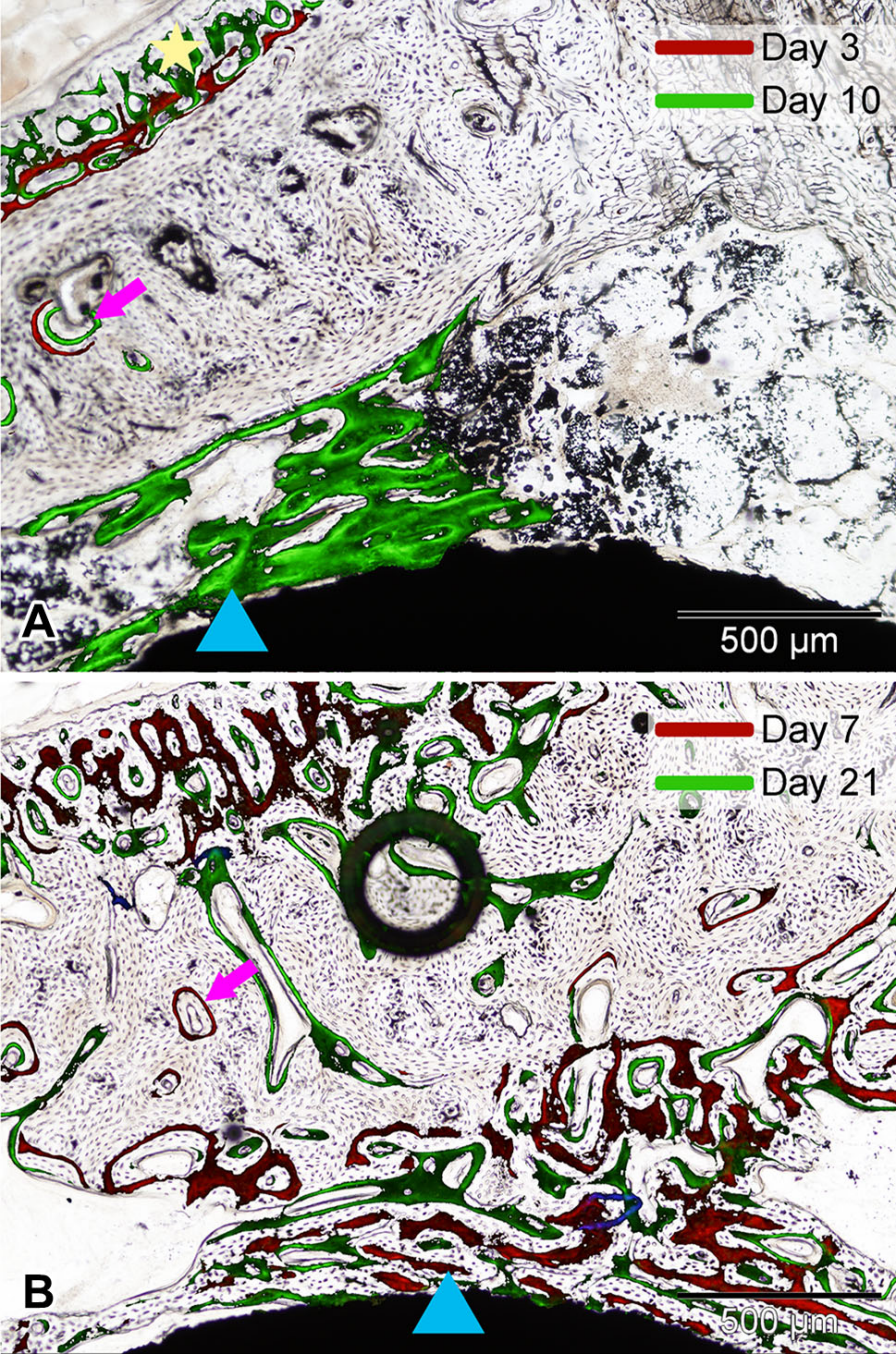
Fluorochrome incorporation was analyzed microscopically, and bright-field and fluorescence pictures were taken from the same areas. The microscopic view of the fluorochrome signals was projected on the corresponding bright-field picture. Shown are representative examples of slides from an animal (Van2 group) injected with fluorochromes on **(A)** Days 3 and 10 and **(B)** Days 7 and 21. All animals showed similar fluorochrome incorporation in newly formed bone in the cortex (pink arrows), around the implant (blue triangles), and in periosteal bone (yellow star). In Illustration B, the dark circle is an air bubble that got incorporated during the embedding process.

All animals showed similar fluorochrome incorporation patterns. Most animals showed mild periosteal bone formation, in some cases already by Day 3 (Fig. 3A). None of the rabbits showed fluorochrome apposition around the implant on Day 3 (Table 1). No differences were found in averaged scores for active bone formation on Day 7 for the Gel group (0.33 ± 0.52) compared with the No Gel group (0.50 ± 0.55; MD, −0.17 95% CI, −0.86 to 0.53; p = 0.78), nor for the Van2 group compared with the No Gel group (0.83 ± 0.41; MD, 0.33; 95% CI, −0.36 to 1.03; p = 0.42), with the numbers available. On Day 21, bone growth around the implant was present in all animals (Table 1; Fig. 3B).

**Table 1:** Timing of bone growth around the implant

Group	Day 3	Day 7	Day 21
No Gel	0 ± 0	0.5 ± 0.5	1 ± 0
Gel	0 ± 0	0.3 ± 0.5	1 ± 0
Van2	0 ± 0	0.8 ± 0.4	1 ± 0

Data are shown as mean ± SD; Van2 = hydrogel loaded with 2% vancomycin; the presence of a fluorochrome on a certain time would result in a score of 1, the absence in a score of 0. For each time, 6 sections were evaluated, except for the No Gel group on Day 3 where 4 sections were evaluated.

### Local and Systemic Inflammation

#### Histopathology

**Fig. 4A–C:**
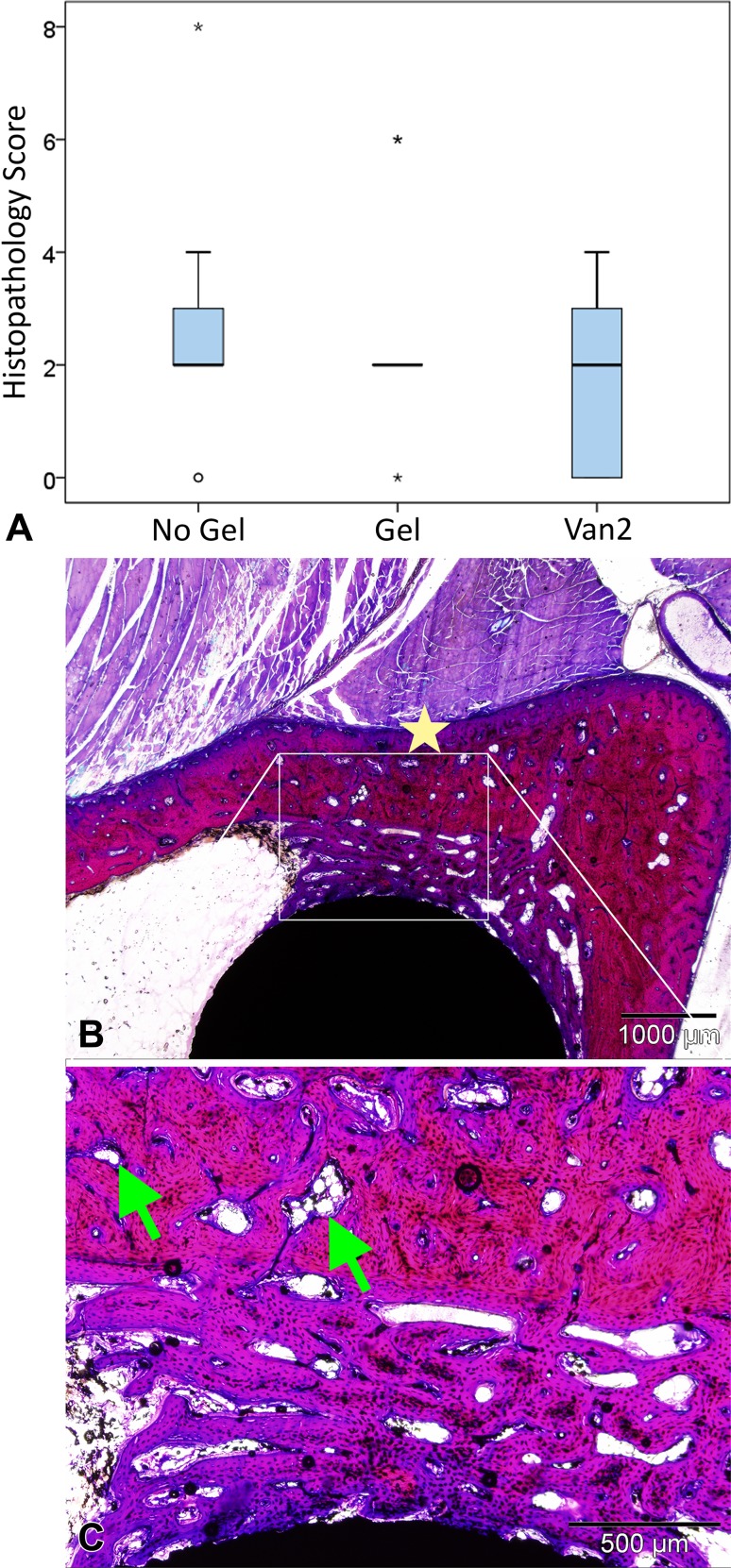
**(A)** Semiquantitative scoring for inflammation parameters was performed on the basic fuchsin and methylene blue stained sections. The data are presented in boxplots with median and range. The circle and stars indicate an outlier and far outliers, respectively. **(B)** A representative microscopic image of a histologic slide is shown together with **(C)** a higher magnification image. All samples showed bone apposition (pink) on the surface of the implant (black). In most animals some Haversian canals were slightly enlarged (green arrows), and a mild periosteal reaction (yellow star) could be observed.

Microscopic analysis of the histologic slides showed few signs of inflammation. The scores in the No Gel group (2.33 ± 1.67) did not differ, with the numbers available, from those of the Gel control group (3.17 ± 1.59; MD, 0.83; 95% CI, −0.59 to 2.26; p = 0.31), nor did the Van2 group (2.5 ± 1.24; MD, 0.17; 95% CI, −1.26 to 1.59; p = 0.95) (Fig. 4A). In general, the Haversian canals were slightly enlarged and there was a mild periosteal reaction observed in all groups (Fig. 4B–C). The similarities in histologic appearance of the groups was confirmed by the histopathology scores for inflammation that ranged from 0 to 56, with 0 representing no inflammation and 56 representing severe inflammation.

### Hematology

**Fig. 5A–G:**
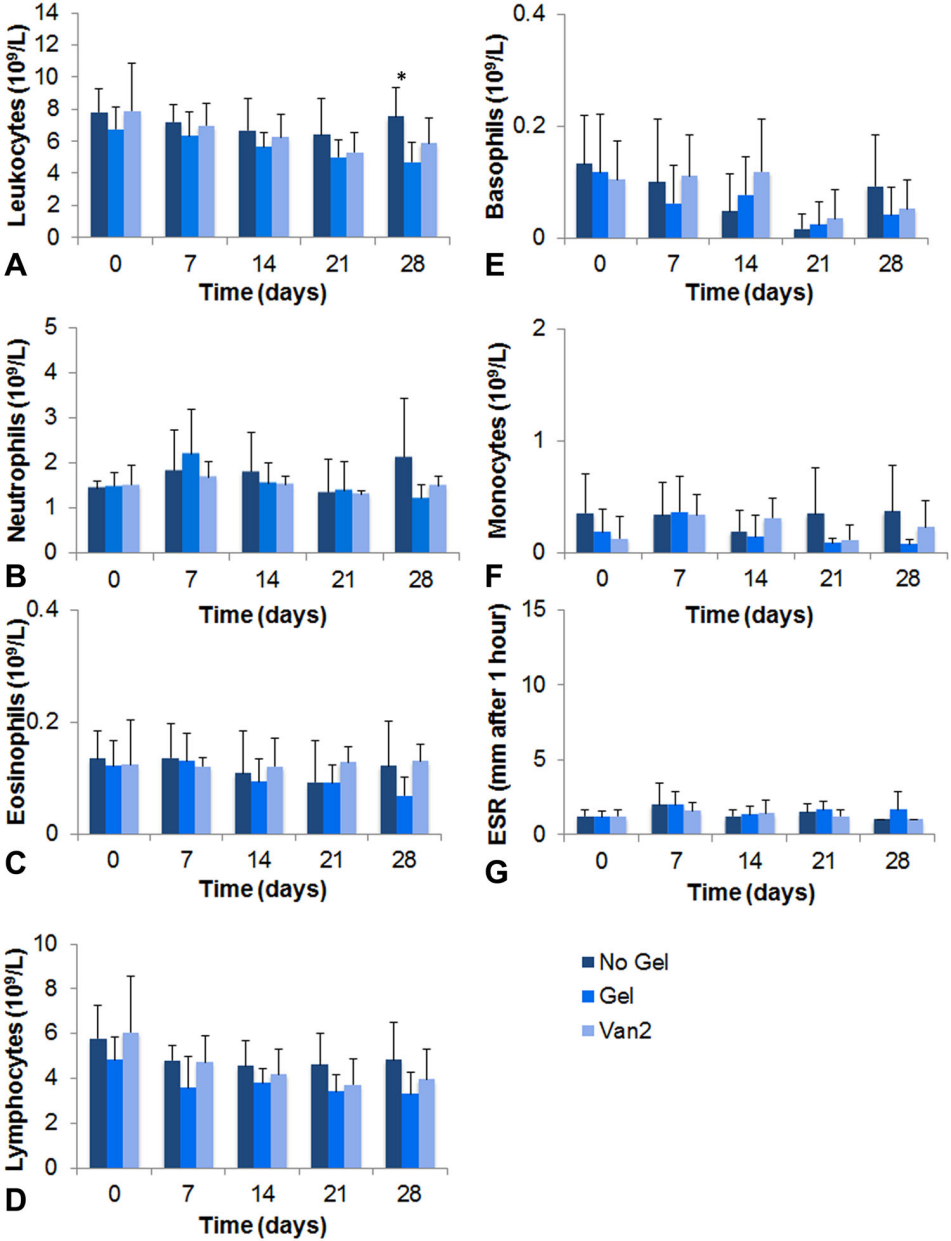
Blood values were measured preoperatively, and weekly during the study. Total **(A)** leukocyte, **(B)** neutrophil, **(C)** eosinophil, **(D)** lymphocyte, **(E)** basophil, and **(F)** monocyte counts, and the **(G)** erythrocyte sedimentation rate (ESR) for all three groups are shown. Data are shown as mean and SD. *p < 0.05 compared with the No Gel control group, at each time.

The hydrogel coating did not result in a hematologic reaction based on the parameters we measured (Fig. 5). No differences in total leukocyte count, ESR, and neutrophil, monocyte, eosinophil, basophil, and lymphocyte counts were present between the No Gel or Van2 groups compared with the Gel control group, with the numbers available. The only exception was the leukocyte count between the No Gel (7.52 ± 1.81 × 10^9^/L) and Gel groups on Day 28 ((4.70 ± 1.21 × 10^9^/L; MD, −2.48; 95% CI, −4.80 to −0.17; p = 0.04)).

## Discussion

Currently, numerous approaches for locally applying antibacterial agents are being researched for prophylaxis of infection for uncemented implants [5, 14, 17, 18]. As bone apposition at the implant surface allows for increased stability of these implants, local antibiotic coatings should not interfere with this process. Therefore, we asked: does a hydrogel or hydrogel loaded with vancomycin (1) interfere with bone apposition and timing of bone deposition near the implant surface; and (2) induce a local or systemic inflammatory reaction as determined by inflammation around the implant and hematologic parameters? In this study, we showed that the tested hydrogel, either unloaded or loaded with 2% vancomycin, did not interfere with bone apposition and timing of bone deposition near the implant surface, and did not induce inflammation around the implant or a systemic inflammatory reaction in a rabbit tibial intramedullary rod model. Only slight changes in morphologic features of the bone were observed, including a mild periosteal reaction and minimally enlarged Haversian canals. These changes might be a reaction to the presence of the implant as these were observed in all groups.

This study had several limitations. First, this was a relatively small animal study, especially for proving the absence of an effect of the hydrogel on bone growth. In previous work with this product, histomorphometric evaluations showed no differences in cortical bone thickness between the hyaluronic acid-based hydrogel and HYALGAN^®^ hydrogel application in a rabbit femur [7]. However, to show the clinical safety of using a hydrogel as a local carrier of antibiotics for orthopaedic implants, this product will need to be validated in a more robust way, for example, a clinical safety study. Second, 28 days followup seems a rather early time to evaluate bone formation. However, at a later time most bone apposition near the implant surface might be near completion which eliminates the possibility of finding differences. In all cases, bone formation around the implant was observed at Day 21, as has been shown by the fluorochrome labels. Therefore, we chose Day 28 as the end-point of the study. Third, the implant model used in this study does not involve a press-fit situation for the implant. Therefore, no conclusions can be drawn regarding stability of the implant.

High local concentrations of antibiotics may be toxic for osteoblasts and could impair bone growth [2, 4]. This would be an unwanted side effect for a local carrier for use in uncemented orthopaedic implants, as optimal bone deposition around the implant is needed for implant stability. In all animals, comparable bone apposition near the implant was observed. At Day 28, the empty hydrogel (59.97%) and the 2% vancomycin-loaded hydrogel (56.12%) showed similar levels of bone volume fraction percentages as the group with uncoated implants (48.65%). Furthermore, all animals showed active bone formation around the implant by Day 21. These results suggest that, in this animal model, the hydrogel, either empty or loaded with 2% vancomycin, does not impair bone formation and that 2% vancomycin was an acceptable concentration to be applied locally.

Inflammation may compromise bone development and delay bone remodeling [1, 9]. None of the animals showed severe signs of inflammation according to the results of the grading system of Vogely et al. [24]. Only slight changes in morphologic features of the bone were observed, including a mild periosteal reaction and minimally enlarged Haversian canals. These changes might be a reaction to the presence of the implant as these were observed in all groups. In addition, there were no meaningful differences in blood values during the study period. These findings suggest that the hydrogel does not induce an inflammation.

One of the potential benefits of using a hydrogel for local delivery of agents is the flexibility in the choice of the functional agent, which possibly can provide personalized antibacterial prophylaxis. Especially for uncemented implants, it would be interesting to explore the possibilities of adding osteoinductive or osteoconductive components next to the antibacterial agents to further improve the bone-implant interface of uncemented implants. A combination of the bone-inducing molecule recombinant human BMP-2 and the antibiotic teicoplanin loaded in the synthetic, degradable polymer poly(D,L-lactic acid)-p-dioxanone-polyethylene glycol resulted in controlled release of teicoplanin for up to 14 days, and critical-sized parietal cranial bone defects in rats were consistently filled with new-formed bone after implantation [16].

An interesting property of hydrogels is that they can be adapted to create a material with specific characteristics that might improve the functionality, for example thermoreversibility, which means that the gels are liquid at lower temperatures and gelate at higher temperatures. In this manner, the hydrogel could be easily syringeable, which allows for easy application, and after gelation the loaded antibiotics could be released in a controlled manner [15, 23]. The versatility of hydrogels and the possibilities to adapt a hydrogel with specific characteristics makes it an interesting candidate for use as a local carrier for agents of interest. Future research could provide more knowledge regarding the ideal properties of a local hydrogel coating for infection prophylaxis and the choice of agents to be loaded in a hydrogel.

In the current animal study, we found that DAC^®^ hydrogel could be applied as a coating on titanium implants without impairing bone formation near the implant surface or inducing an inflammatory reaction. Future clinical safety studies may provide additional evidence that this hydrogel does not hinder bone formation and prove the safety of this product. Antibiotic-loaded hydrogel may be a valuable option to offer local protection of orthopaedic implants from bacterial colonization.
